# Bis(1-ethyl-3-methyl­imidazolium) 3,6-diselanyl­idene-1,2,4,5-tetra­selena-3,6-diphospha­cyclo­hexane-3,6-di­selen­olate

**DOI:** 10.1107/S1600536813020308

**Published:** 2013-07-31

**Authors:** Jason A. Cody, Grant C. B. Alexander, Catherine Guillot-Deudon

**Affiliations:** a Lake Forest College, 555 N. Sheridan Rd, Lake Forest, IL 60045, USA; b Institut des Matériaux Jean Rouxel (IMN), UMR 6502 CNRS-Université de Nantes, 2 rue de la Houssinière, BP 32229, 44322 Nantes Cedex 03, France

## Abstract

In the title compound, 2C_6_H_11_N_2_^+^·P_2_Se_8_^2−^ or [EMIM]_2_P_2_Se_8_ (EMIM = 1-ethyl-3-methyl­imidazolium), the anions, located about inversion centers between EMIM cations, exhibit a cyclo­hexane-like chair conformation. The cations are found in columns along the *a* axis, with centroid–centroid distances of 3.8399 (3) and 4.7530 (2) Å. The observed P—Se distances and Se—P—Se angles agree with other salts of this anion.

## Related literature

For similar seleno­phosphate compounds, see: Biswas *et al.* (2010 ▶); Lin *et al.* (2012 ▶). For ionothermal reactions in room-temperature ionic liquids, see: Morris (2009 ▶); Parnham & Morris (2007 ▶); Cody *et al.* (2012 ▶). For the preparation of EMIM(BF_4_), see: Egashira *et al.* (2006 ▶). For the structure of the P_2_Se_8_^2−^ anion, see: Zhao *et al.* (1992 ▶); Rotter *et al.* (2008 ▶). For π–π inter­actions between imidazolium cations, see: Wilkes & Zaworotko (1993 ▶).
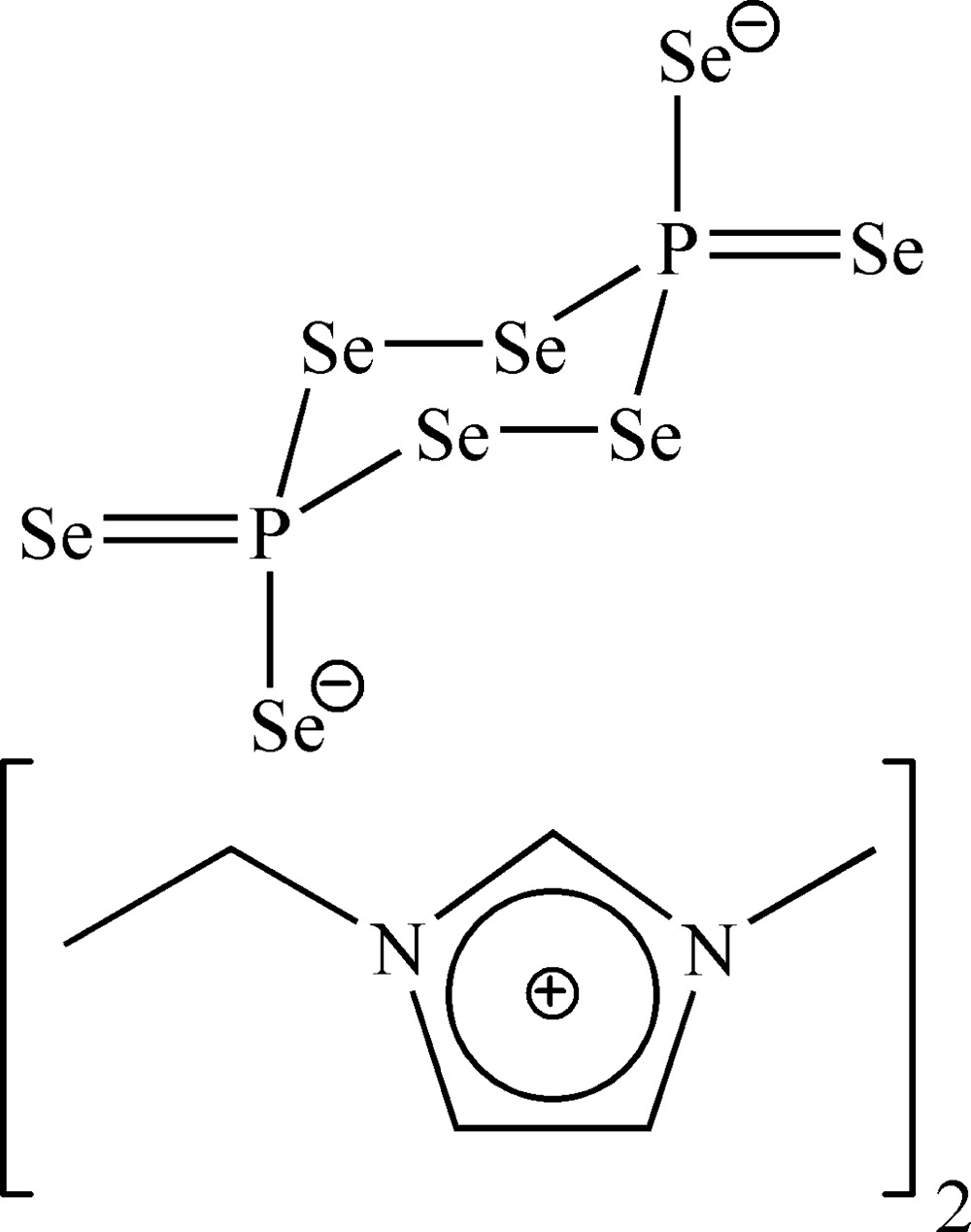


## Experimental

### 

#### Crystal data

2C_6_H_11_N_2_^+^·P_2_Se_8_^2−^
*M* *_r_* = 915.96
Triclinic, 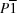

*a* = 7.8885 (4) Å
*b* = 9.3783 (4) Å
*c* = 9.8039 (5) Å
α = 110.390 (3)°
β = 96.395 (4)°
γ = 102.992 (5)°
*V* = 648.00 (5) Å^3^
*Z* = 1
Mo *K*α radiation
μ = 11.41 mm^−1^
*T* = 293 K
0.19 × 0.07 × 0.03 mm

#### Data collection

Nonius KappaCCD diffractometer
Absorption correction: Gaussian [*JANA2006* (Petříček *et al.*, 2006 ▶) and *X-SHAPE* (Stoe & Cie, 1998 ▶)] *T*_min_ = 0.204, *T*_max_ = 0.754
22118 measured reflections
3719 independent reflections
2622 reflections with *I* > 2σ(*I*)
*R*_int_ = 0.072

#### Refinement

*R*[*F* ^2^ > 2σ(*F* ^2^)] = 0.029
*wR*(*F* ^2^) = 0.063
*S* = 1.02
3719 reflections
118 parameters
H-atom parameters constrained
Δρ_max_ = 0.47 e Å^−3^
Δρ_min_ = −0.55 e Å^−3^

### 

Data collection: *COLLECT* (Hooft, 2009 ▶); cell refinement: *COLLECT*; data reduction: *COLLECT*; program(s) used to solve structure: *SHELXS97* (Sheldrick, 2008 ▶); program(s) used to refine structure: *SHELXL97* (Sheldrick, 2008 ▶); molecular graphics: *DIAMOND* (Brandenburg & Putz, 2012 ▶); software used to prepare material for publication: *SHELXL97*.

## Supplementary Material

Crystal structure: contains datablock(s) global, I. DOI: 10.1107/S1600536813020308/nc2314sup1.cif

Structure factors: contains datablock(s) I. DOI: 10.1107/S1600536813020308/nc2314Isup2.hkl

Supplementary material file. DOI: 10.1107/S1600536813020308/nc2314Isup3.mol

Supplementary material file. DOI: 10.1107/S1600536813020308/nc2314Isup4.cml

Additional supplementary materials:  crystallographic information; 3D view; checkCIF report

## Figures and Tables

**Table:** 

P1—Se4	2.1104 (8)
P1—Se3	2.1334 (8)
P1—Se1	2.2794 (9)
P1—Se2^i^	2.2809 (8)
Se1—Se2	2.3442 (5)

**Table:** 

Se4—P1—Se3	122.19 (4)
Se4—P1—Se1	113.49 (4)
Se3—P1—Se1	100.04 (3)
Se4—P1—Se2^i^	113.90 (4)
Se3—P1—Se2^i^	100.49 (3)
Se1—P1—Se2^i^	104.32 (3)
P1—Se1—Se2	102.89 (2)
P1^i^—Se2—Se1	102.37 (2)

Symmetry code: (i) 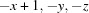.

**Fig. 1.**
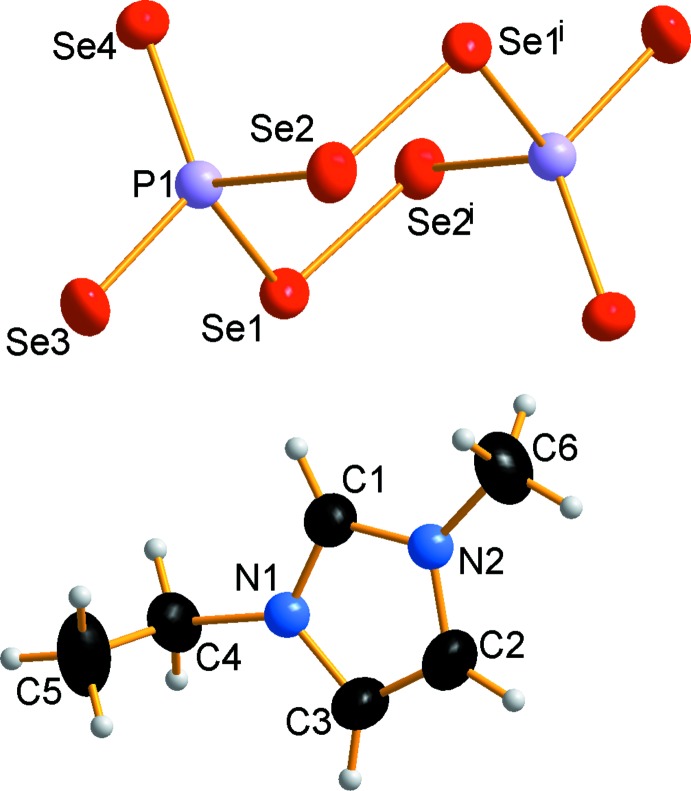
Molecular structure and atomic labeling scheme for the title compound. Thermal elipsoids are shown at the 50% level. Symmetry code for the generation of equivalent atoms: i = 1 - x, -y, -z.

**Fig. 2.**
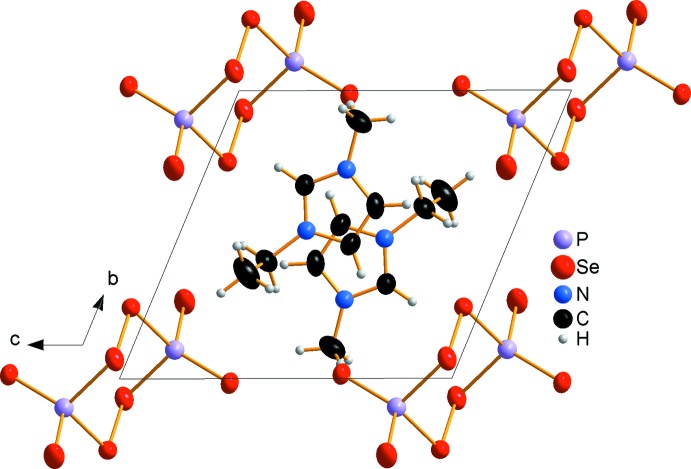
A packing diagram of the title compound showing the column of EMIM cations along the a axis.

## supplementary crystallographic information

### Comment

Thiophosphate and selenophosphate compounds are sought for their interesting and fine-tunable electronic properties (Lin *et al.*, 2012). As new synthetic methods are often needed to gain access to new compounds with interesting properties, ionothermal reactions of thio- and selenophosphates were explored. Recently, room-temperature ionic liquids have received much interest for the preparation of inorganic materials (Morris, 2009; Parnham & Morris, 2007; Cody, *et al.*, 2012).

The structure of the anion in the title compound was first reported by Zhao *et al.* (1992) and the EMIM cation is well known. All interatomic distances and angles found in the current study (Fig. 1) are within normal ranges (Rotter *et al.*, 2008). Although the EMIM cations are found in columns in the title compound, the centroid-to-centroid distances of 3.8399 (3) Å and 4.7530 (2) Å (Fig. 2) indicate only weak pi-pi interactions (Wilkes & Zaworotko, 1993).

### Experimental

The title compound was prepared by the literature method (Cody *et al.*, 2012) for ionothermal synthesis of related sulfur compounds. A total mass of 125 mg of the elements with a stoichiometry of Ni: 4P: 16Se was ground together in a glove box and then placed in a Pyrex tube. An aliquot of 1.25 ml of the ionic liquid EMIMBF_4_, prepared according to the literature (Egashira, *et al.*, 2006), was added to the tube in a glove bag. The tube was then evacuated and sealed. The reaction mixture was heated at 150 °C for 96 h and then slowly cooled to room temperature at a rate of 0.5 °C/min. The tube was opened, the product mixture was filtered, and individual crystals were selected for analysis by hand. The products included black powder, large red blocks, and small yellow plates. The latter were both the title compound; the color difference is attributed to absorption effects from the thickness of the crystals. Although elemental nickel was included in the reaction mixture, it was not observed in the isolated crystalline products.

### Refinement

All H atoms were positioned with idealized geometry and were refined isotropically with *U*_iso_(H) = 1.2 *U*_eq_(C) (1.5 for methyl H atoms) using a riding model with C—H = 0.93 Å for aromatic, 0.97 Å for methylene and 0.96 Å for methyl H-atoms.

### Crystal data

**Table:**

2C_6_H_11_N_2_^+^·P_2_Se_8_^2^^−^	*Z* = 1
*M**_r_* = 915.96	*F*(000) = 424
Triclinic, *P*1	*D*_x_ = 2.347 Mg m^−^^3^
Hall symbol: -P 1	Mo *K*α radiation, λ = 0.71073 Å
*a* = 7.8885 (4) Å	Cell parameters from 3719 reflections
*b* = 9.3783 (4) Å	θ = 6.5–30.0°
*c* = 9.8039 (5) Å	µ = 11.41 mm^−^^1^
α = 110.390 (3)°	*T* = 293 K
β = 96.395 (4)°	Plate, yellow
γ = 102.992 (5)°	0.19 × 0.07 × 0.03 mm
*V* = 648.00 (5) Å^3^	

### Data collection

**Table:**

Nonius KappaCCD diffractometer	3719 independent reflections
Radiation source: fine-focus sealed tube	2622 reflections with *I* > 2σ(*I*)
Unknown monochromator	*R*_int_ = 0.072
ω scans	θ_max_ = 30.0°, θ_min_ = 6.5°
Absorption correction: gaussian [JANA2006 (Petříček *et al.*, 2006) and *X-SHAPE* (Stoe & Cie, 1998)]	*h* = −10→11
*T*_min_ = 0.204, *T*_max_ = 0.754	*k* = −13→13
22118 measured reflections	*l* = −13→13

### Refinement

**Table:**

Refinement on *F*^2^	Primary atom site location: structure-invariant direct methods
Least-squares matrix: full	Secondary atom site location: difference Fourier map
*R*[*F*^2^ > 2σ(*F*^2^)] = 0.029	Hydrogen site location: inferred from neighbouring sites
*wR*(*F*^2^) = 0.063	H-atom parameters constrained
*S* = 1.02	*w* = 1/[σ^2^(*F*_o_^2^) + (0.0252*P*)^2^ + 0.2292*P*] where *P* = (*F*_o_^2^ + 2*F*_c_^2^)/3
3719 reflections	(Δ/σ)_max_ < 0.001
118 parameters	Δρ_max_ = 0.47 e Å^−^^3^
0 restraints	Δρ_min_ = −0.55 e Å^−^^3^

### Special details

**Table:**

Experimental. A set of 280 frames were collected with a rotation of 2° per frame and an exposure time of 190 s; the crystal to detector distance was 25.00 mm. Refinements were done with the *SHELXTL97* (Sheldrick, 2008) software package; absorption correction was made with the program Jana2006 (Petricek *et al.*, 2006) using the program *X-SHAPE* (Stoe & Cie, 1998).
Geometry. All e.s.d.'s (except the e.s.d. in the dihedral angle between two l.s. planes) are estimated using the full covariance matrix. The cell e.s.d.'s are taken into account individually in the estimation of e.s.d.'s in distances, angles and torsion angles; correlations between e.s.d.'s in cell parameters are only used when they are defined by crystal symmetry. An approximate (isotropic) treatment of cell e.s.d.'s is used for estimating e.s.d.'s involving l.s. planes.
Refinement. Refinement of *F*^2^ against ALL reflections. The weighted *R*-factor *wR* and goodness of fit *S* are based on *F*^2^, conventional *R*-factors *R* are based on *F*, with *F* set to zero for negative *F*^2^. The threshold expression of *F*^2^ > σ(*F*^2^) is used only for calculating *R*-factors(gt) *etc*. and is not relevant to the choice of reflections for refinement. *R*-factors based on *F*^2^ are statistically about twice as large as those based on *F*, and *R*- factors based on ALL data will be even larger.

### Fractional atomic coordinates and isotropic or equivalent isotropic displacement parameters (Å^2^)

**Table:**

	*x*	*y*	*z*	*U*_iso_*/*U*_eq_	
P1	0.62769 (11)	−0.10004 (9)	0.13027 (9)	0.03455 (17)	
Se1	0.38273 (4)	−0.24755 (3)	−0.05425 (4)	0.04208 (9)	
Se2	0.21381 (4)	−0.06847 (4)	−0.03916 (4)	0.04234 (9)	
Se3	0.77764 (6)	−0.26777 (5)	0.09996 (4)	0.05519 (11)	
Se4	0.56642 (5)	0.01558 (4)	0.33743 (4)	0.04503 (10)	
N1	0.2827 (4)	0.4858 (3)	0.3773 (3)	0.0413 (6)	
N2	0.2005 (3)	0.2740 (3)	0.4218 (3)	0.0422 (6)	
C1	0.2281 (4)	0.3279 (4)	0.3154 (4)	0.0415 (7)	
H1	0.2122	0.2661	0.2148	0.050*	
C2	0.2379 (5)	0.4007 (5)	0.5542 (4)	0.0530 (9)	
H2	0.2298	0.3961	0.6465	0.064*	
C3	0.2884 (5)	0.5333 (4)	0.5270 (4)	0.0525 (9)	
H3	0.3210	0.6372	0.5965	0.063*	
C4	0.3182 (5)	0.5911 (4)	0.2952 (4)	0.0486 (8)	
H4A	0.3597	0.5382	0.2068	0.058*	
H4B	0.4115	0.6869	0.3572	0.058*	
C5	0.1558 (6)	0.6340 (6)	0.2513 (6)	0.0735 (13)	
H5A	0.1832	0.7026	0.1986	0.110*	
H5B	0.1156	0.6877	0.3388	0.110*	
H5C	0.0641	0.5395	0.1883	0.110*	
C6	0.1325 (5)	0.1069 (4)	0.3983 (5)	0.0606 (10)	
H6A	0.1248	0.0980	0.4922	0.091*	
H6B	0.2114	0.0496	0.3527	0.091*	
H6C	0.0165	0.0634	0.3345	0.091*	

### Atomic displacement parameters (Å^2^)

**Table:**

	*U*^11^	*U*^22^	*U*^33^	*U*^12^	*U*^13^	*U*^23^
P1	0.0454 (4)	0.0353 (4)	0.0258 (4)	0.0132 (3)	0.0065 (3)	0.0144 (3)
Se1	0.0560 (2)	0.03274 (15)	0.03188 (17)	0.00568 (13)	0.00294 (14)	0.01201 (13)
Se2	0.03936 (17)	0.05217 (19)	0.03963 (19)	0.00924 (13)	0.00866 (14)	0.02474 (16)
Se3	0.0774 (3)	0.0598 (2)	0.0454 (2)	0.0411 (2)	0.01621 (19)	0.02586 (19)
Se4	0.0639 (2)	0.04233 (17)	0.02882 (17)	0.01506 (15)	0.01340 (15)	0.01247 (14)
N1	0.0472 (15)	0.0410 (14)	0.0361 (15)	0.0097 (11)	0.0087 (12)	0.0172 (12)
N2	0.0435 (14)	0.0439 (15)	0.0431 (16)	0.0089 (11)	0.0079 (12)	0.0239 (13)
C1	0.0461 (18)	0.0424 (16)	0.0338 (17)	0.0084 (13)	0.0078 (14)	0.0147 (14)
C2	0.063 (2)	0.066 (2)	0.036 (2)	0.0179 (18)	0.0138 (17)	0.0268 (19)
C3	0.071 (2)	0.0468 (18)	0.0329 (18)	0.0121 (17)	0.0126 (17)	0.0101 (16)
C4	0.054 (2)	0.0459 (18)	0.052 (2)	0.0092 (15)	0.0174 (17)	0.0277 (17)
C5	0.065 (3)	0.095 (3)	0.095 (4)	0.030 (2)	0.025 (2)	0.069 (3)
C6	0.064 (2)	0.051 (2)	0.075 (3)	0.0095 (17)	0.013 (2)	0.038 (2)

### Geometric parameters (Å, º)

**Table:**

P1—Se4	2.1104 (8)	C2—C3	1.345 (5)
P1—Se3	2.1334 (8)	C2—H2	0.9300
P1—Se1	2.2794 (9)	C3—H3	0.9300
P1—Se2^i^	2.2809 (8)	C4—C5	1.489 (5)
Se1—Se2	2.3442 (5)	C4—H4A	0.9700
Se2—P1^i^	2.2809 (8)	C4—H4B	0.9700
N1—C1	1.332 (4)	C5—H5A	0.9600
N1—C3	1.370 (4)	C5—H5B	0.9600
N1—C4	1.477 (4)	C5—H5C	0.9600
N2—C1	1.327 (4)	C6—H6A	0.9600
N2—C2	1.366 (4)	C6—H6B	0.9600
N2—C6	1.463 (4)	C6—H6C	0.9600
C1—H1	0.9300		
			
Se4—P1—Se3	122.19 (4)	C2—C3—H3	126.6
Se4—P1—Se1	113.49 (4)	N1—C3—H3	126.6
Se3—P1—Se1	100.04 (3)	N1—C4—C5	111.4 (3)
Se4—P1—Se2^i^	113.90 (4)	N1—C4—H4A	109.3
Se3—P1—Se2^i^	100.49 (3)	C5—C4—H4A	109.3
Se1—P1—Se2^i^	104.32 (3)	N1—C4—H4B	109.3
P1—Se1—Se2	102.89 (2)	C5—C4—H4B	109.3
P1^i^—Se2—Se1	102.37 (2)	H4A—C4—H4B	108.0
C1—N1—C3	108.8 (3)	C4—C5—H5A	109.5
C1—N1—C4	125.1 (3)	C4—C5—H5B	109.5
C3—N1—C4	126.0 (3)	H5A—C5—H5B	109.5
C1—N2—C2	108.5 (3)	C4—C5—H5C	109.5
C1—N2—C6	125.1 (3)	H5A—C5—H5C	109.5
C2—N2—C6	126.3 (3)	H5B—C5—H5C	109.5
N2—C1—N1	108.3 (3)	N2—C6—H6A	109.5
N2—C1—H1	125.9	N2—C6—H6B	109.5
N1—C1—H1	125.9	H6A—C6—H6B	109.5
C3—C2—N2	107.8 (3)	N2—C6—H6C	109.5
C3—C2—H2	126.1	H6A—C6—H6C	109.5
N2—C2—H2	126.1	H6B—C6—H6C	109.5
C2—C3—N1	106.7 (3)		

Symmetry code: (i) −*x*+1, −*y*, −*z*.
